# Quantifying the probability of a successful marine bioinvasion due to source‐destination risk factors

**DOI:** 10.1002/ece3.10984

**Published:** 2024-03-19

**Authors:** Mimi W. Tzeng, Lisa Floerl, Jessica Schattschneider, Oliver Floerl, Andrew Jeffs, Anastasija Zaiko

**Affiliations:** ^1^ Institute of Marine Science University of Auckland Auckland New Zealand; ^2^ Cawthron Institute Nelson New Zealand; ^3^ Tasman District Council Richmond New Zealand; ^4^ Land Water People Ltd Christchurch New Zealand; ^5^ Sequench Ltd Nelson New Zealand

**Keywords:** environmental similarity, global commercial shipping, marine bioinvasions, probability of establishment, probability of introduction, voyage‐related risk factors

## Abstract

The increasing spread of marine non‐indigenous species (NIS) due to the growth in global shipping traffic is causing widespread concern for the ecological and economic impacts of marine bioinvasions. Risk management authorities need tools to identify pathways and source regions of priority concern to better target efforts for preventing NIS introduction. The probability of a successful NIS introduction is affected by the likelihood that a marine species entrained in a transport vector will survive the voyage between origin and destination locations and establish an independently reproducing population at the destination. Three important risk factors are voyage duration, range of environmental conditions encountered during transit and environmental similarity between origin and destination. In this study, we aimed for a globally comprehensive approach to assembling quantifications of source‐destination risk factors from every potential origin to every potential destination. To derive estimates of voyage‐related marine biosecurity risk, we used computer‐simulated vessel paths between pairs of ecoprovinces in the Marine Ecoregions Of the World biogeographic classification system. We used the physical length of each path to calculate voyage duration risk and the cross‐latitudinal extent of the path to calculate voyage path risk. Environmental similarity risk was based on comparing annual average sea surface temperature and salinity within each ecoprovince to those of other ecoprovinces. We derived three separate sets of risk quantifications, one each for voyage duration, voyage path and environmental similarity. Our quantifications can be applied to studies that require source‐destination risk estimates. They can be used separately or combined, depending on the importance of the types of source‐destination risks that might be relevant to particular scientific or risk management questions or applications.

## Graphical abstract

The probability of a successful marine bioinvasion is affected by the likelihood that a potentially invasive marine species entrained in a transport vector (e.g., cargo ships) will survive the voyage between origin and destination locations, and establish an independently reproducing population at the destination. Three important risk factors are voyage duration, range of environmental conditions encountered during transit and environmental similarity between origin and destination. In this study, we aimed for a globally comprehensive approach to assembling quantifications of source‐destination risk factors from every potential origin to every potential destination.

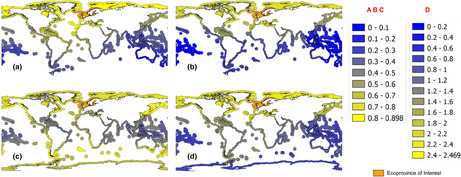

## 1 INTRODUCTION

Marine bioinvasions are occurring at a global scale mainly due to the incidental translocation of marine non‐indigenous species (NIS) by maritime vessels (Ruiz et al., 1997). Transport of marine NIS occurs predominantly via internal ballast water tanks or as biofouling on the submerged external surfaces of the hull (Drake & Lodge, 2007; Hewitt et al., 2009; Molnar et al., 2008; Ruiz et al., 2000). The most common type of vessel available for marine NIS translocations is cargo vessels of the global commercial shipping fleet (Hulme, 2009).

The probability of a successful marine bioinvasion via shipping traffic is affected by the likelihood that marine NIS will survive the journey between the origin and recipient locations (Verling et al., 2005; Zaiko et al., 2020), i.e., the probability of introduction (Lodge et al., 2016; Seebens et al., 2013). During a sea voyage, living organisms travelling on a vessel are likely to experience stressful conditions that increase their mortality rate (Zaiko et al., 2020). For example, hull fouling organisms might experience low food availability (Schimanski et al., 2016), temperature and salinity changes (Chan et al., 2015; Edmiston et al., 2021), exposure to water turbulence and weather effects depending on their location on the hull (Coutts et al., 2010) and at high latitudes there might be scouring by sea ice (Hughes & Ashton, 2017; Leidenberger et al., 2015). Ballast water organisms might experience food and oxygen depletion, temperature changes and increases in concentration of ammonia and other waste products (Verling et al., 2005; Zaiko et al., 2020).

The two main voyage‐related risk factors that affect the probability of introduction are duration and path. Voyage duration determines the period that organisms must endure stressful conditions and is directly related to the geographic distance between source and destination locations. Assuming that cargo vessels travel at similar speeds, greater distances will mean longer voyage durations, presumed higher mortality rates and therefore reduced residual biosecurity risk by the time the vessel reaches its destination. Meanwhile, the voyage path can affect the range of environmental conditions experienced by potential marine NIS carried on the vessel, in particular, if the vessel crosses climatic zones, i.e., the magnitude of change in sea surface temperature (Chan et al., 2015; Edmiston et al., 2021). Voyage paths that include more cross‐latitude movement will experience greater temperature changes and are therefore likely to have a higher mortality rate and lower biosecurity risk (Chan et al., 2015; Edmiston et al., 2021).

In addition to the voyage‐related risk factors during transit, the degree of environmental similarity between origin and recipient locations affects the probability that marine NIS that survived the journey will also survive to successfully establish an independently reproducing population at the destination following their introduction, i.e., the probability of establishment (Lodge et al., 2016; Seebens et al., 2013). A common approach for assessing environmental similarity between two locations is to calculate an environmental distance measure, using abiotic factors such as water temperature and salinity (Keller et al., 2011; Tzeng, 2022c). Environmental similarity quantifications represent relative rather than absolute measures of risk. A low environmental distance value would indicate high environmental similarity and thus greater likelihood for marine NIS to survive the environmental conditions of the recipient location. The approach is especially useful when considering the potential movement of a large number of different species that are not individually assessed for their specific environmental requirements (Barry et al., 2008).

Past voyage‐related risk factors have been estimated empirically using specific sea voyages, such as latitudinally across climate zones (Zaiko et al., 2020) or across gradients in both temperature and salinity (Edmiston et al., 2021), with impacts on organisms both in ballast water tanks (Zaiko et al., 2020) and on the hull (Edmiston et al., 2021). Likewise, past environmental distance calculations have largely been in relation to a specific location, such as the North American Great Lakes (Keller et al., 2011), New Zealand (Floerl et al., 2013), or the Madeira Archipelago, Portugal (Castro et al., 2022). In this study, we aim for a more globally comprehensive approach of assembling quantifications of source‐destination risk factors from every potential origin to every potential destination, which can be applied at the scope of any of the above mentioned studies as well as to future studies that require source‐destination risk estimates.

## 2 METHODS

To define the geographical locations from which source‐destination risks can be quantified, we used the *Marine Ecoregions Of the World* (MEOW) biogeographic classification system (Spalding et al., 2007). MEOW divides the coastal, nearshore and shelf areas of the world into a nested hierarchy of 232 ecoregions, 62 ecoprovinces and 12 ecorealms. Each ecoregion is ecologically distinct from the others, encompassing an area of a size typical to the geographic range of the life histories and ecological processes of marine species with a sedentary adult phase. Similarly, each ecoprovince is of a scale for more mobile or dispersive species. Ecorealms are large, broad areas of distinct biota at higher taxonomic classification levels. Detailed global maps of the MEOW system are available in Spalding et al. (2007).

The ecoprovince level of the hierarchy was chosen as the most appropriate resolution to use for a global‐scale assessment and for testing the approach on generalised scenarios of cross‐regional NIS transfer, i.e., without a particular coastline or species of concern being in focus. We also considered the future applicability of the derived outputs for the trade‐based biosecurity risk model currently operating at this hierarchy level (Lenzen et al., 2023).

### 2.1 Voyage‐related risk factors: Duration and path

The global movement patterns of cargo vessels are highly complex. Some vessels make multiple stops along a circulating path as part of a regular route, while others will change course and destination mid‐transit due to changes in market demand for their particular cargo (Kaluza et al., 2010; Stopford, 2009a). In some cases, multiple possible routes are available. However, the conservative approach in risk assessment is the one that maximises potential risk, which in this case assumes minimum voyage duration. Therefore, we assumed that all shipping voyages occur along the shortest, most direct paths between geographic locations, taking into account the locations of landmasses and traversable canals (e.g., Suez, Panama) and not considering interim stops elsewhere, detours due to severe weather, or avoidance of dangerous waters or piracy. Since shipping routes are generally established with economics in mind, the geographically shortest sea route is most likely to be chosen whenever feasible. Although we acknowledge that other obstacles (e.g., political situations or safety concerns) might affect the route selection, these are not considered in our approach as they are context‐dependent and not consistent factors.

MEOW ecoprovinces are irregularly shaped geographical areas along the world's coastlines. To generate voyage paths between each pair of ecoprovinces, it was first necessary to define the endpoints for the paths as point locations. Centroid point coordinates were generated for each ecoprovince using the Calculate Geometry Attributes tool in ArcGIS Pro (version 2.7.3). Auto‐generated centroids located on land were manually repositioned to the nearest coastal location. The centroid for Ecoprovince 1 (Arctic), which was auto‐generated near the North Pole, was manually repositioned closer to northern Europe – assuming more intense maritime shipping in this area based on the current marine traffic records (https://www.marinetraffic.com) and near‐future projections (Melia et al., 2017; Stephenson & Smith, 2015).

The Subantarctic Islands and Continental High Antarctic (Ecoprovinces 59 and 61) were excluded from the analysis because they encircle the entire continent of Antarctica and any reasonably located centroids would be in the middle of Antarctica. They are also unlikely to experience high levels of shipping traffic at present, although this may change with climate change (Duffy et al., 2017; Hughes & Ashton, 2017). Missing data from these locations are indicated with NA in the data matrices containing the eventual results.

Voyage paths were generated between each pair of ecoprovince centroids in a multi‐step process (Schattschneider et al., 2022) using the R package ‘gdistance’ (van Etten, 2017), which calculates the shortest line distances between point locations without intersecting land.

Polygons representing the world's oceans were modified to allow paths to pass through narrow shipping canals (Suez and Panama) and waterways (e.g., Singapore Strait, Turkish Straits), then rasterised (WGS84 geographic coordinate system), with 1 assigned to water areas and ‘no data’ to land areas. The northern extent of the raster was sufficient to allow path generation through Arctic waters if required. A raster cell size of 0.05 degrees (approximately 5 km at the equator) provided reasonable computational time (a few days) but high enough resolution to force paths around, rather than over, narrower land bridges.

The raster image of the world's oceans was converted into a transition matrix or ‘neighbour graph’ of cell centres (nodes), where the matrix values represent transition or conductance from one node to another. Each node connects with eight orthogonal and diagonal neighbouring nodes. Connections with four, eight and 16 neighbours were tested and eight was found to provide the best outcome with respect to tracking around coastlines and retaining raster information.

A geometric correction was applied to the transition matrix to resolve distortions caused by differences in distance between diagonal or orthogonal neighbours and between west–east connections in a longitude‐latitude grid where meridians are closer together near the poles. The function used for correcting the transition matrix divides each conductance value by the distance between cell centres. Distances are calculated as great‐circle distances (‘geoCorrection’ function with the type argument set to ‘c’). The least cost distance analysis used in this project assumes an equal cost to move between the ‘water’ cells of the raster.

The shortest over‐water distances between each pair of ecoprovince centroids were then calculated using the least cost distance analysis, i.e., by applying the ‘CostDistance’ function to the geo‐corrected transition matrix. The resulting physical distance values are presented in meters.

To confirm that the resulting paths tracked around coastlines and not over land, mapped lines representing the paths were generated and visually checked. This process resulted in multiple line segments per path, however, the lengths of combined lines matched the output from the ‘CostDistance’ function, thereby validating that the ‘CostDistance’ function produced the intended output.

The minimum and maximum latitude for each line segment were calculated using bounding boxes within the R package ‘sf’ (Pebesma, 2018). Where more than one line segment per path existed, output data were summarised to select the highest maximum latitude and lowest minimum latitude per path. Minimum and maximum latitude, which are in decimal degrees with negative values for the Southern Hemisphere, were then used to calculate cross‐latitude distance (Tzeng, 2022b). For paths that were entirely in one hemisphere, the cross‐latitude distance was calculated as the difference between minimum and maximum latitude. We assumed that the temperature gradient reverses direction on either side of the equator, therefore, for paths that cross the equator, the cross‐latitude distance used was the absolute value of the latitude farthest from the equator.

### 2.2 Environmental distance between source and destination

Environmental distance data between MEOW ecoprovinces were obtained from Tzeng (2022c), where environmental distances were calculated from seawater temperature (Locarnini et al., 2019) and salinity (Zweng et al., 2019) data from World Ocean Atlas (WOA, Boyer et al., 2018) using a generalised linear model approach as described in Keller et al. (2011) and MEOW polygons adapted from Spalding et al. (2007) by The Nature Conservancy (2016). The resulting environmental distance values have no intrinsic value in and of themselves, but are mainly a comparative measure to values between other location pairs and therefore have no units.

### 2.3 Calculation of standardised risk for each risk factor

The three types of distance values, i.e., environmental distance values, physical distance values and cross‐latitude distance values, were all in different units, i.e., unitless, meters and decimal degrees, respectively. To allow for direct comparison among distance values, they were converted into standardised risk values using the following procedure. For each type of distance value, the risk decreases as distance increases, so the distances were rescaled from 0 to 1 by standardising to the maximum value on an inverted scale. To ensure that the maximum distance (minimum relative risk) would still have a risk above 0, the maximum distance was rounded up to the next highest integer. All other distance values were then divided by the rounded‐up maximum value and the result was subtracted from 1 to arrive at the risk value. The equation used for this procedure was risk = 1 − (distance/[rounded up maximum distance]). In addition, the risk for distances of 0 was set to 0 to indicate that an ecoprovince is not a risk to itself.

Environmental distance values were converted to environmental similarity risk. Physical distance values were converted to voyage duration risk. Cross‐latitude distances were converted to voyage path risk. All risk values range from 0 to 1. An estimate of total source‐destination risk for each pair of ecoprovinces was calculated assuming equal risk from each factor, by adding together environmental similarity risk, voyage duration risk and voyage path risk, resulting in values ranging from 0 to 3.

### 2.4 Visualisations based on specific ecoprovinces of interest

Heat maps were created to visualise the global distribution of source‐destination risk. Using QGIS 3.22, the MEOW polygons downloaded from Data Basin were joined to the distance datasets by their corresponding ecoprovince numbers. A gradient of yellow to blue was selected to indicate low to high values for each type of distance.

Heat maps were created to showcase the physical distance and cross‐latitudinal extent of a voyage path for one high‐latitude and one low‐latitude ecoprovince (2. Northern European Seas and 43. Tropical East Pacific). For environmental distances, heat maps were obtained from Tzeng (2022c) to showcase two high‐latitude ecoprovinces (1. Arctic and 2. Northern European Seas) and two low‐latitude ecoprovinces (12. Caribbean and 43. Tropical East Pacific); the latter two were chosen for being on opposite sides of an isthmus.

To visualise global patterns of relative risk, we chose the Northern European Seas (Ecoprovince 2) as an ecoprovince of interest. This area is well established as both a source and recipient location for many marine NIS. Examples include the European green crab, *Carcinus maenas*, which has well‐established invasion histories in eastern and western North America and South Africa (Grosholz & Ruiz, 1996) and the clubbed ascidian, *Styela clava*, which originated in the Sea of Japan and began invading the northern European seas in the 1950s (Lützen, 1999). Heat maps were created for environmental similarity risk, voyage duration risk, voyage path risk and estimated total source‐destination risk.

### 2.5 Comparison of estimated risk to empirical events

We attempted to assess the effectiveness of our source‐destination risk estimates by comparing the values for the Northern European Seas (Ecoprovince 2) to empirical data on NIS introductions available from AquaNIS (AquaNIS, 2021; Olenin et al., 2014). Although the number of NIS cannot be treated as a direct measure of the threat posed to the native biota, environment or economies (Molnar et al., 2008), higher numbers of NIS generally indicate active introduction pathways operating in the region. With an accumulation of introductions, the likelihood increases for the establishment of an invasive species and adverse effects on recipient ecosystems (Pires‐Teixeira et al., 2021; Simberloff & von Holle, 1999). Given that quantitative information on the impacts of marine NIS on a regional level is very scarce or non‐existent, we considered the number of recorded NIS as a proxy for bioinvasion pressure in the region. We hypothesised that an increase in estimated risk should be correlated to an increase in the number of recorded marine NIS. The available empirical dataset includes the number of marine NIS recorded to have been introduced by maritime vessels between 1900 and 2010 to the North Sea, Baltic Sea and the Celtic‐Biscay Shelf and identifies their likely source location. These areas are part of the Large Marine Ecosystem (LME) biogeographic classification system of marine environments (Sherman & Alexander, 1986), corresponding to 22, 23 and 24, respectively.

For each marine NIS introduced to the Northern European Seas, we matched the area where it was reported to have originated, as identified by the LME system, to the closest corresponding ecoprovince from the MEOW system. For single ecoprovinces that spanned multiple LMEs, we summed marine NIS counts across the LMEs. For single LMEs that spanned multiple ecoprovinces, we generally omitted the marine NIS counts due to the increased uncertainty of averaging risk values across larger areas. However, in one case two adjacent LMEs and two adjacent ecoprovinces had similar overlap in covered areas among the classification systems and could be combined; in this case, we summed the marine NIS counts across the LMEs and averaged the various types of source‐destination risk values between ecoprovinces. Once we determined the successful matches between classification systems, we applied linear regression to derive the coefficient of determination to relate the marine NIS counts and the estimated risk values.

## 3 RESULTS

The final dataset (Tzeng, 2022a) consists of six matrices, each of which contains one of the following variables associated with each possible pair of ecoprovinces: environmental distance (from Tzeng, 2022c), environmental similarity risk, physical distance (m), voyage duration risk, cross‐latitude distance (decimal degrees) and voyage path risk.

### 3.1 Voyage‐related risk factors: Duration and path

Physical distances ranged from a minimum of 855 km between northern New Zealand (Ecoprovince 53) and southern New Zealand (Ecoprovince 54), to a maximum of 21,936 km between the Warm Temperate Northwest Atlantic, i.e., the northern Gulf of Mexico and coastal southeastern United States (Ecoprovince 6) and Java Transitional, i.e., the southside coastal area of Indonesia near Jakarta (Ecoprovince 27). Cross‐latitude distances had a minimum of 0.14° between the Tropical Southwestern Pacific (Ecoprovince 35) and Southeast Polynesia (Ecoprovince 40) and a maximum of 84.92° between the Arctic (Ecoprovince 1) and Java Transitional (Ecoprovince 27).

**FIGURE 1:**
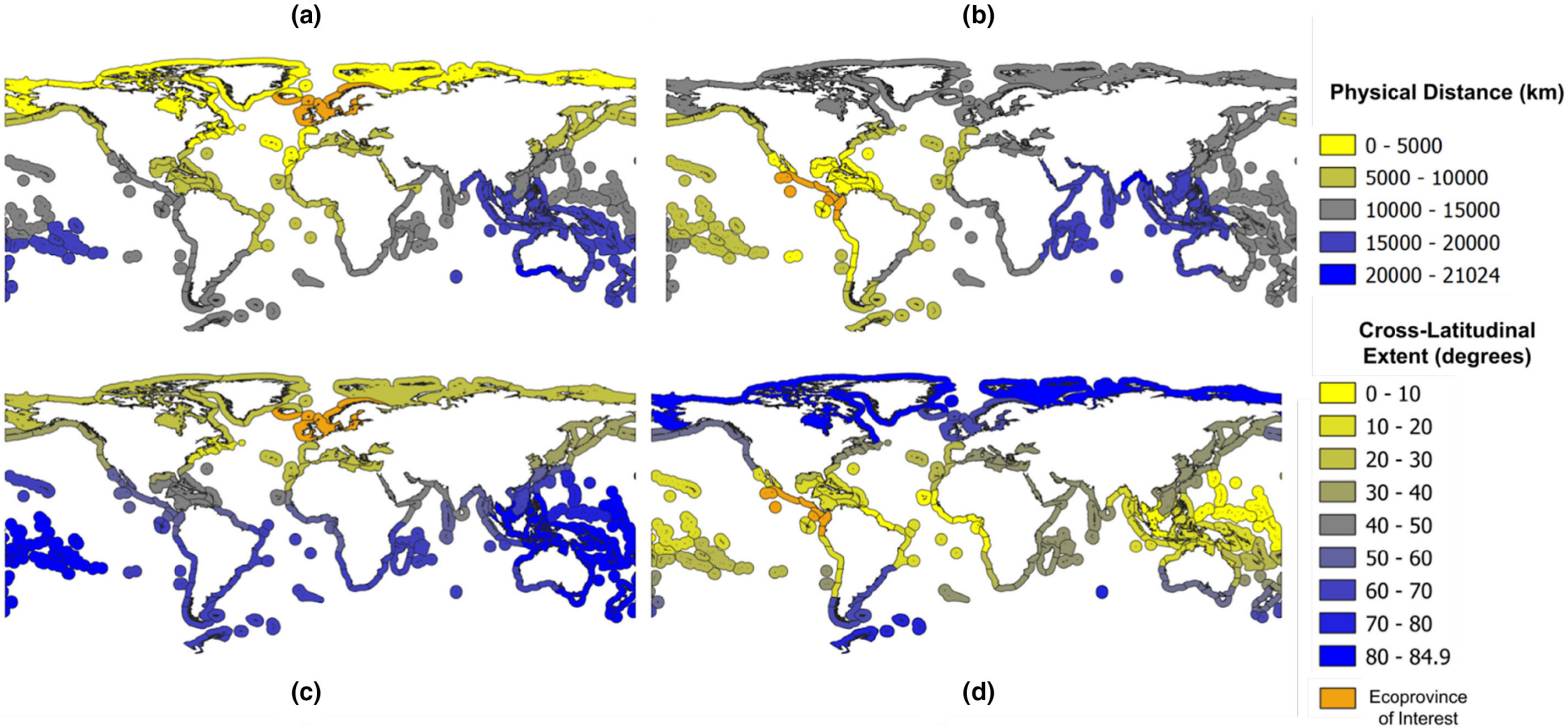
Heat maps showing physical distance (km) and cross‐latitude extent (degrees) of the voyage path to or from the Northern European Seas (Ecoprovince 2) and the Tropical East Pacific (Ecoprovince 43), ranging from yellow (low distance) to blue (high distance), with the origin ecoprovince highlighted in orange. (a) Physical distance relative to Ecoprovince 2. (b) Physical distance relative to Ecoprovince 43. (c) Cross‐latitude distance relative to Ecoprovince 2. (d) Cross‐latitude distance relative to Ecoprovince 43.

For the Northern European Seas (Ecoprovince 2), the patterns for physical and cross‐latitude distances are similar, with low distances for nearby areas in the surrounding waters and higher distances to the Southern Hemisphere and the Indian Ocean (Figure 1a,c). For the Tropical East Pacific (Ecoprovince 43), the Panama Canal allows areas of the Atlantic Ocean to be physically closer than they would be otherwise, while the cross‐latitude extent of voyage paths is lowest near the equator and highest towards the poles, as might be expected (Figure 1b,d).

### 3.2 Environmental distance between source and destination

**FIGURE 2:**
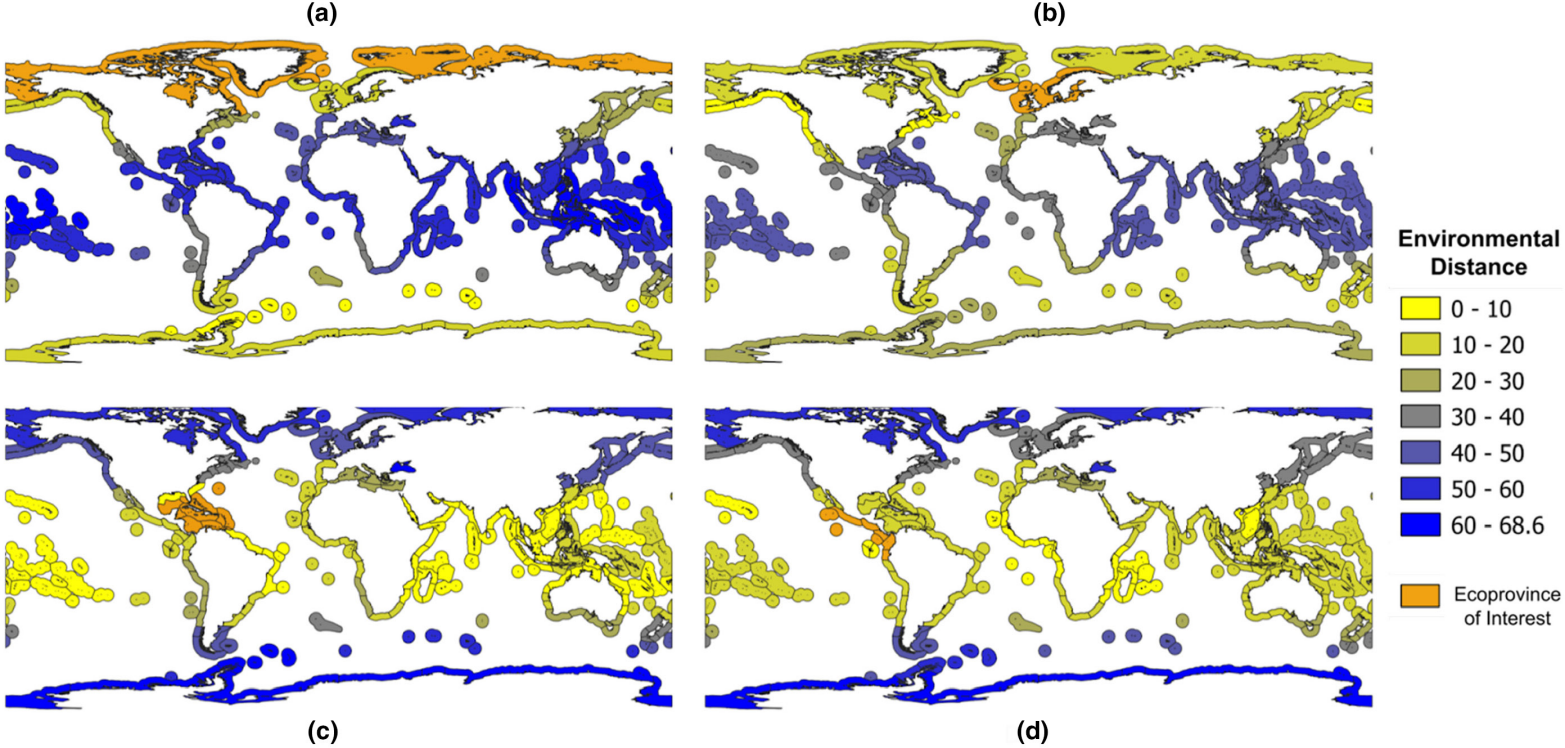
Heat maps showing environmental distance relative to (a) the Arctic (Ecoprovince 1), (b) the Northern European Seas (Ecoprovince 2), (c) the Caribbean (Ecoprovince 12) and (d) the Tropical East Pacific (Ecoprovince 43). Environmental distances range from yellow (low distance) to blue (high distance), with the origin ecoprovince highlighted in orange. The figure is sourced from Tzeng (2022c).

Environmental distance values range from 0 to 73.02, where 0 is the distance of an ecoprovince from itself (Figure 2, Tzeng, 2022c). As might be expected, ecoprovinces at similar latitudes tend to be the least environmentally distant. For example, the Arctic (Ecoprovince 1) is least environmentally distant from the Magellanic, Subantarctic Islands, Scotia Sea and Continental High Antarctic (Ecoprovinces 48, 59, 60, 61) with values ranging from 3.38 to 10.67 (Figure 2a). In contrast, the Arctic is most environmentally distant from areas along the equator such as the Eastern and Western Coral Triangle and Central Polynesia (Ecoprovinces 30, 31, 39), with values ranging from 61.50 to 63.27 (Figure 2a). The Northern European Seas (Ecoprovince 2) show a similar pattern to the Arctic. Both the Arctic and the Northern European Seas are environmentally more similar to the Subantarctic Islands than to coastal Antarctica (Figure 2b).

Along the same latitude, salinity differences may play a larger role than temperature, since geographically nearby ecoprovinces are not necessarily the most similar to each other. For example, looking at either side of Central America, the Caribbean (Ecoprovince 12) is least distant from Somali/Arabian (Ecoprovince 19) with a value of 2.56, while the Tropical East Pacific (Ecoprovince 43) is least distant from the Gulf of Guinea (Ecoprovince 17) with a value of 3.33 (Figure 2c,d). Indeed the Caribbean is more distant from the Gulf of Guinea (value of 12.75) compared to the Tropical East Pacific, even though they are on opposite sides of the Atlantic Ocean and also more distant from the Tropical East Pacific (13.77), which is geographically on the other side of an isthmus.

### 3.3 Comparison of estimated risk to empirical events: A closer look at the Northern European Seas (Ecoprovince 2)

**FIGURE 3:**
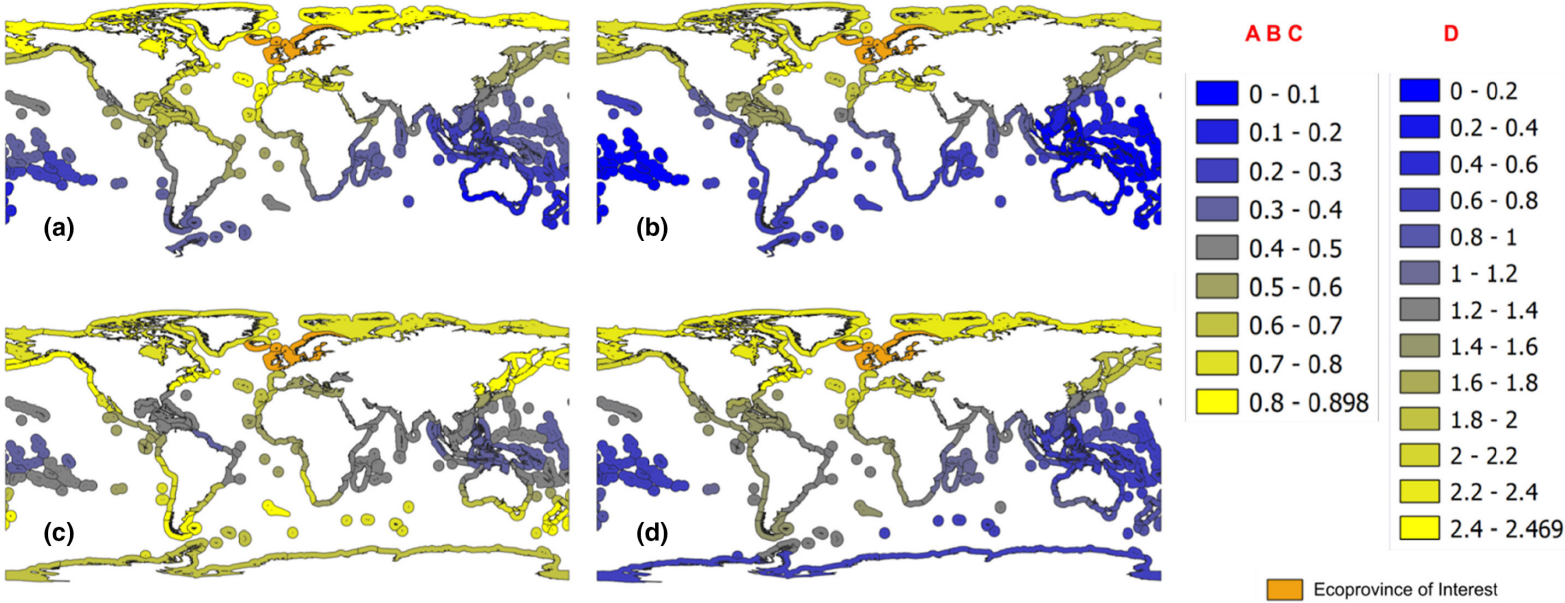
Heat maps showing marine biosecurity risk relative to the Northern European Seas (Ecoprovince 2), on a scale from dark blue (low risk) to yellow (high risk). (a) Voyage duration risk. (b) Voyage path risk. (c) Environmental similarity risk. (d) Combined risk: risks were combined by adding them together. The scales for a, b and c are 0.0–1.0, while for combined risks (d) it ranges from 0.0 to 3.0. Voyage duration and path data (a and b) did not include two Antarctic ecoprovinces (59 and 61).

The heat maps showing relative source‐destination risks for the Northern European Seas (Figure 3) are similar to the heat maps for the corresponding distance types (Figures 1a,c and 2b). In general, risk is reduced as geographic distance increases away from the Northern European Seas, or if the voyage path passes through areas of higher temperature. However, the risk is higher with closer environmental similarity of the destination. When each risk type is weighted equally, combined risk in the Northern Hemisphere reflects the same risk patterns as the individual risk types (i.e., environmental similarity, voyage duration and voyage path), while for the Southern Hemisphere, there is a general decrease in risk (Figure 3d).

We identified 12 LMEs and MEOW ecoprovinces where marine NIS originated and spread to the Northern European Seas (Table 1). Seven of the matched areas had only one recorded marine NIS introduced to the Northern European Seas. The relative risk from those areas ranged from 0.001 (voyage path risk from Australia) to 0.836 (voyage duration risk from the Lusitanian) on a scale from 0 to 1. These seven ‘singleton’ matches were omitted, because they potentially confounded any correlations among the remaining matches.

**TABLE 1:** Estimated source‐destination risk relative to the Northern European Seas (MEOW Ecoprovince 2) compared to the number of marine NIS introduced to the North Sea (LME 22), Baltic Sea (LME 23), or Celtic‐Biscay Shelf (LME 24).

Origin LME of Marine NIS	MEOW Ecoprovince corresponding to origin LME	Environmental similarity risk	Voyage duration risk	Voyage path risk	Combined risk	Number of introduced Marine NIS
7/8. Northeast U.S. Continental Shelf, Scotian Shelf	5. Cold Temperate NW Atlantic	0.886	0.780	0.803	2.469	15
25. Iberian Coastal	3. Lusitanian	0.644	0.836	0.680	2.161	1
26. Mediterranean Sea	4. Mediterranean Sea	0.558	0.749	0.711	2.018	5
48/50. Yellow Sea, Sea of Japan/East Sea	8. Cold Temperate NW Pacific	0.837	0.545	0.576	1.958	3
62/A2. Black Sea, Caspian Sea	7. Black Sea	0.471	0.661	0.711	1.843	31
5/6. Gulf of Mexico, Southeast U.S. Continental Shelf	6/12. Warm Temperate, Tropical NW Atlantic	0.442	0.637	0.550	1.629	10
12. Caribbean Sea	12. Tropical NW Atlantic	0.418	0.649	0.518	1.586	1
11. Pacific Central‐American Coastal	43. Tropical East Pacific	0.523	0.514	0.371	1.407	1
17. North Brazil Shelf	13. North Brazil Shelf	0.399	0.636	0.339	1.373	1
47. East China Sea	9. Warm Temperate NW Pacific	0.555	0.431	0.362	1.349	1
42. Southeast Australian Shelf	56. SE Australian Shelf	0.748	0.123	0.001	0.872	1
39. North Australian Shelf	32. Sahul Shelf	0.384	0.229	0.001	0.614	1

*Note*: The listed origin LMEs are the most likely source locations of the marine NIS as determined in AquaNIS. The listed ecoprovinces of the MEOW system are the closest match to the origin LMEs. The table has been organised by descending amounts of combined risk.

**FIGURE 4:**
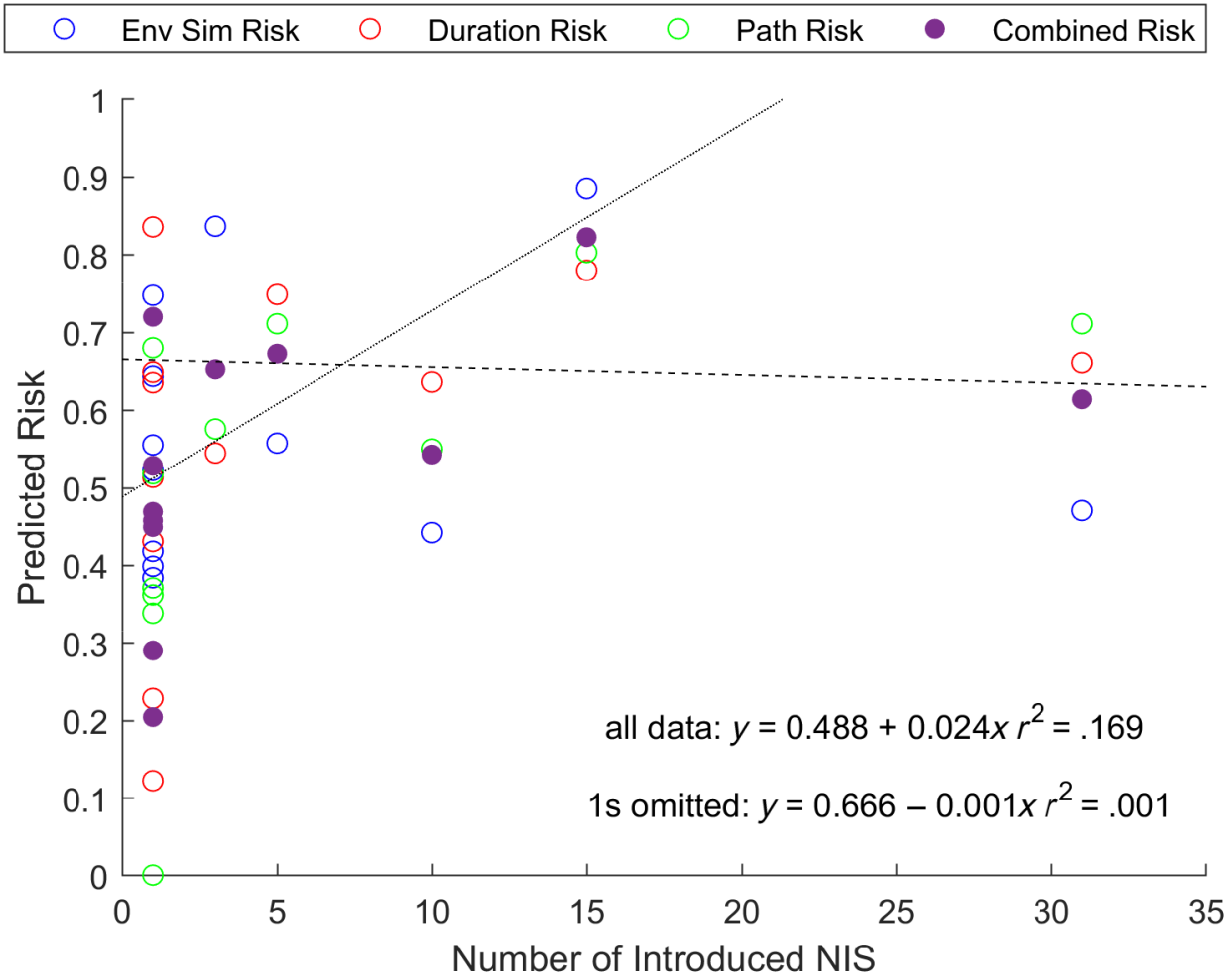
Estimated source‐destination risks for marine NIS relative to the Northern European Seas (Ecoprovince 2) compared to the number of marine NIS introduced to the North Sea (LME 22), Baltic Sea (LME 23) or Celtic‐Biscay Shelf (LME 24). The combined risk was divided by 3 to allow plotting on the same scale as the individual risks. Linear regression lines and equations are for combined risk data, with a solid line representing all data and a dashed line representing data with ‘1’s omitted.

The remaining five matches showed no clear increase in relative source‐destination risk with the number of marine NIS introduced to the Northern European Seas from other parts of the world (Figure 4). Although the size of the dataset is insufficient for statistical analyses and the result of the ground truthing assessment is inconclusive, the overall trend suggests that source areas of multiple marine NIS have higher estimated risk than source areas of singular marine NIS, i.e., the combined risk is greater than 1.6 (Table 1).

## 4 DISCUSSION

### 4.1 Overview and potential uses

The primary goal of this study was to develop a method for creating global maps of relative marine biosecurity risk between specific source and destination location pairs that can be attributed to maritime vessel traffic. We quantified three types of relative source‐destination risk estimates among pairs of MEOW ecoprovinces: environmental similarity risk, voyage duration risk and voyage path risk. For environmental similarity, the relative risk was based on average temperature and salinity differences between the two locations. For the voyage‐related risk factors, we simulated the shortest, most direct path between the geographic centres (centroids) of each pair of ecoprovinces and derived the total distance and cross‐latitudinal extent of each path. We then created maps from the resulting dataset that focused on four of the 62 possible ecoprovinces as risk origins (Figures 1, 2, 3) as examples of how the data can be visualised.

The resulting global risk matrices can be used separately or combined, using various weighting schemes to suit a variety of research questions. For example, for species‐specific studies, tolerance ranges or overall hardiness of species might be taken into account to adjust the influence of voyage path or environmental similarity on their mortality rate during transport or their ability to establish at the destination (e.g., Chan et al., 2015; Edmiston et al., 2021). For marine NIS that have a stress‐resistant dormant stage, voyage path or duration are less relevant and can be substantially downweighed or disregarded (Gray et al., 2005; Radzikowski, 2013).

The source‐destination risk estimates can support several practical biosecurity management challenges. For example, biosecurity authorities throughout the world typically assess the compliance of incoming vessels to national or international policies (e.g., Clarke et al., 2017; Trindade Castro et al., 2018), such as the Ballast Water Management Convention (IMO, 2004), the Brazilian Maritime Standard for ballast water management (NORMAM‐20, 2014), the Australian biofouling management requirements for international vessel arrivals (Australian Government, 2019) or the Craft Risk Management Standard for biofouling (New Zealand Government, 2018). Our risk estimates could be used to optimise resource allocation for inspections and risk assessments by prioritising the selection of vessels that have travelled from or through high‐risk locations.

Our risk matrices can also support surveillance programs for the early detection of high‐risk marine NIS that are known to have potentially high impacts within their invasive range, such as New Zealand's National Marine High‐Risk Site Surveillance (MHRSS) (e.g., Woods et al., 2020) and the Australian Marine Pest Monitoring Strategy (Arthur et al., 2015; Australian Government, 2010). Consideration of NIS targeted via such programs can partly be informed by our source‐destination risk framework, following appropriate species‐specific parameterisation. Our risk estimates can also be used to help refine lists of target species for surveillance by considering vessel arrivals from high‐risk origin locations.

With source‐destination risk estimates available between each pair of ecoprovinces, it becomes possible to examine vessel‐based source‐destination risk between pairs of nations. The coastal areas of each nation pair could be matched to determine which of their corresponding ecoprovinces are most likely to be engaged in trade. When combined with economic analysis techniques such as multi‐region input–output (MRIO) modelling, this approach could contribute towards predicting the global movement patterns of marine biosecurity risk species based on the global trade of commodities (e.g., Lenzen et al., 2023).

### 4.2 Comparison of risk estimates to empirical data

AquaNIS (AquaNIS, 2021) is an online database of aquatic (marine, brackish and freshwater) NIS that have been introduced primarily to Europe and neighbouring areas, although updates are in active progress for other parts of the world. Available information includes introduction histories, taxonomy, biological traits, impacts, known recipient regions and possible source regions. Data within the database can be subset in various ways, for example by introduction method (i.e., vessels) or current status (i.e., abundant vs. rare vs. extinct). The most comprehensive coverage areas within AquaNIS are the North Sea and the Baltic Sea, which are both within the bounds of MEOW Ecoprovince 2 (Northern European Seas).

Unfortunately, although AquaNIS contains a wealth of highly detailed qualitative information about introduced species and introduction events, it is not designed for high‐volume quantitative analyses, such as a direct region‐by‐region comparison with our global scale source‐destination risk estimates. For example, the copepod *Acartia tonsa* is recorded to have been introduced to the Baltic Sea, North Sea or Celtic‐Biscay Shelf eight times in total, with seven of them from adjacent regions in the Indian Ocean. It is unclear from the provided data whether the records indicate seven separate introductions, one from each region or one introduction that could have come from any of the seven regions. Relative marine biosecurity risk levels among the regions cannot be determined from this type of empirical data and in retrospect upon discovering these nuances in the AquaNIS data, a direct region‐by‐region comparison of the derived risk estimates from this project to the empirical data from AquaNIS is not possible. Unfortunately, no other coverage areas within AquaNIS are as comprehensive as the North and Baltic Seas and no other open sources of empirical data on marine bioinvasions are as comprehensive and publicly accessible as AquaNIS, though smaller databases exist (e.g., NEMESIS for the United States, Fofonoff et al., 2018). While a more effective ground‐truthing comparison of our risk estimates would be beneficial, a rigorous in‐depth validation is not possible at this time with currently available databases.

Other risk factors can affect the numbers of successful marine NIS introductions, beyond the factors examined for the source‐destination risk estimates of this study, which can also confound the comparison results. Some examples are the time of year as it relates to the reproductive seasons of specific marine NIS and propagule pressure, i.e., the frequency and magnitude of marine NIS arrivals (Carlton, 1996; Lockwood et al., 2005). The latter is strongly influenced by the movement patterns of cargo vessels (Ceballos‐Osuna et al., 2021), i.e., the number and size of vessels transporting trade goods between various source‐destination locations (Verna et al., 2021), which is correlated to the strength of trade connections between regions. A complete risk assessment strategy would need to account for propagule pressure as well as the source‐destination risks (Verling et al., 2005). Similarly, the ballast water and biofouling management practices of individual cargo vessels will cause the mortality rate of marine NIS aboard the vessels to vary and therefore vary their probability of introduction at the destination locations. With an increase in the number of cargo vessels that implement ballast water management practices in accordance with the Ballast Water Management Convention (IMO, 2004) and the Biofouling Guidelines (IMO, 2011, 2023), the probability of introduction from high‐risk locations is also likely to decrease over time.

Even with all of the existing limitations, origin locations that were sources of multiple marine NIS have an observed general trend of higher risk values than origin locations that were sources for only one marine NIS (Table 1). Therefore, the overall approach used to produce source‐destination risk estimates in this study has the potential for robustness.

### 4.3 Future opportunities

The framework for source‐destination biosecurity risks quantification and risk estimates presented in this study provides a foundation for various potential applications within the domain of biosecurity risk assessment, including prioritisation of management actions, horizon scanning for species or regions posing future risks, modelling global marine NIS spread scenarios and many others. For example, our framework can be readily used to complement a recent model characterising the global patterns of biosecurity risks based on seaborne trade connections between nations (Lenzen et al., 2023). This model utilises extensive long‐term global commodity import–export data, allowing for assessments in both retrospective and forecasting modes and for identifying the riskiest origin region, commodity and ship type combinations. The standardised estimates for voyage‐ and environmental similarities‐related risk factors are critical but currently missing input parameters of this model. The outputs of this study help to fill this gap. Below we provide some additional consideration regarding the useability of the presented approach and the potential for future adjustments depending on specific study requirements or marine biosecurity questions in focus.

Environmental distances have been calculated with as many as 37 variables in the past (Hilliard & Raaymakers, 1997). However, seawater temperature and salinity represent the most important abiotic factors of a marine habitat when generalising across many species (Barry et al., 2008), which formed the rationale for the method used to calculate the environmental distances used in this study (Keller et al., 2011; Tzeng, 2022c). The available code (Tzeng, 2022c) can be customised to include other factors provided in the WOA (Boyer et al., 2018), such as oxygen, phosphate, silicate or nitrate, if the factors are important to the environmental suitability for a particular species of interest.

For voyage duration risks, which were estimated based on the geographic distances of the transit paths of maritime vessels, we assumed that all vessels travel at the same speed. However, different vessel types have different at‐sea speeds, e.g., bulkers and tankers are relatively slow compared to container vessels (Stopford, 2009b; Tzeng et al., 2021). Moreover, higher vessel speeds are correlated with higher mortality rates among hull fouling organisms (e.g., Coutts et al., 2009, 2010), while at the same time correlated with reduced voyage duration and therefore reduced mortality rates among ballast tank organisms (Verling et al., 2005; Zaiko et al., 2020). The relative risk due to voyage duration can be weighted according to vessel type if specific vessel types are of interest (Tzeng et al., 2021), or according to whether a species of interest is primarily carried in ballast water (e.g., the copepod *Acartia tonsa*, Gubanova et al., 2014) or on the hull (e.g., the bryozoan *Bugula neritina*, Cohen, 2011).

For voyage path risks, the range of seawater temperature along the path is a dominant influence when considering stressors experienced by marine organisms in both the ballast water tanks and the hull. However, changes in salinity along the path can also directly affect the mortality rate of hull biofouling organisms, while organisms in the ballast tanks would be unaffected. For example, freshwater NIS can be introduced successfully to remote freshwater systems separated from their native range by oceans, if they are carried in ballast tanks (e.g., the zebra mussel *Dreissena polymorpha*, Nalepa & Schloesser, 2013). Likewise, temperature and salinity conditions within traversable canals can be distinct from the conditions in the oceans to either side. For example, a voyage path that traverses the Panama Canal would be more likely to increase the mortality rate of marine NIS on the hull due to exposure to freshwater in the locks (Menzies, 1968), while a path through the Suez Canal would expose the hull fouling marine NIS to hypersaline conditions. Voyage path risk estimates could be modified to account for salinity in risk assessments that include such situations.

The source‐destination risk factors accounted for in our study can effectively complement other biosecurity risk assessment models (Verling et al., 2005). For example, global shipping network analyses (Kaluza et al., 2010; Muirhead & MacIsaac, 2005; Seebens et al., 2016; Wang et al., 2018; Xu et al., 2014) used for approximating the frequency and magnitude of marine NIS introductions (propagule pressure; Carlton, 1996; Lockwood et al., 2005), would benefit from the inclusion of estimates of source‐destination risk factors. With increasing compliance with ballast water (IMO, 2004) and biofouling management (IMO, 2011, 2023) requirements for international vessel movements, the probability of marine NIS introductions from high‐risk sources will likely decrease over time and the relevant risk matrices could be weighted accordingly, allowing an adaptive management approach.

The source‐destination risk estimates can be modified to account for global climate change, which is expected to warm the polar regions, affect the pattern of shipping routes and increase the rate of successful marine bioinvasions in both the Arctic (e.g., Chan et al., 2018; Ware et al., 2015) and Antarctic (e.g., Duffy et al., 2017; Hughes & Ashton, 2017). An increase in sea surface temperature will increase the environmental suitability for a larger number of marine NIS at the poles (Duffy et al., 2017; Ware et al., 2014), while an increase in shipping traffic will increase propagule pressure (Chan et al., 2015; McCarthy et al., 2022; Miller & Ruiz, 2014; Ware et al., 2015).

For example, for environmental suitability, we calculated our risk estimates for this study based on present‐day seawater temperature and salinity data (Boyer et al., 2018; Tzeng, 2022c), but it is also possible to calculate environmental similarity based on projected future climatic conditions in the coastal environment (Floerl et al., 2013) using model predictions from the IPCC (Bindoff et al., 2019). For the voyage‐related risk estimates, the simulated voyage paths could be reconfigured to allow travel through the Arctic Ocean and the centroid for Ecoprovince 1 (Arctic) could be repositioned near the North Pole rather than closer to the North Atlantic than the North Pacific. Centroids could be manually added for the two Antarctic ecoprovinces (59 and 61), perhaps in proximity to ports in Antarctica.

To develop our source‐destination risk estimate methods, we used the ecoprovince level of the MEOW system. The framework could also be adopted to derive risk estimates at the ecoregion level, which has 232 divisions of the world's coastal areas encompassed within the 62 divisions at the ecoprovince level (Spalding et al., 2007), which would enable research questions to be addressed at a finer scale.

## 5 CONCLUSIONS

Our study provides a comprehensive framework for quantifying marine biosecurity risk between source and destination pairs, focusing on voyage‐related risk factors and environmental similarity. The examination of physical distances, cross‐latitude extents and environmental differences among ecoprovinces allowed us to discern nuanced patterns in relative source‐destination risks. While physical distances and cross‐latitude extents elucidate spatial relationships, environmental similarity plays a significant role in determining the likelihood of successful NIS introductions. The adaptability of our quantifications allows for tailored risk assessments, accommodating species‐specific traits or emphasising specific risk factors pertinent to the transportation or establishment capabilities for particular species of concern. Our approach enables a deeper understanding of vessel‐based source‐destination risks between nations, potentially linking these insights with economic models to predict global movement patterns of biosecurity risk species based on the global trade of commodities.

In summary, our framework lays a solid foundation for diverse biosecurity risk assessments and management strategies, providing valuable insights into the complex dynamics of marine bioinvasions and guiding proactive measures to protect marine ecosystems and economies worldwide.

## AUTHOR CONTRIBUTIONS

**Mimi W. Tzeng:** Conceptualization (equal); data curation (lead); formal analysis (lead); investigation (lead); methodology (equal); project administration (supporting); software (equal); validation (lead); visualization (lead); writing – original draft (lead); writing – review and editing (lead). **Lisa Floerl:** Formal analysis (supporting); methodology (equal); software (equal); visualization (supporting); writing – original draft (supporting); writing – review and editing (supporting). **Jessica Schattschneider:** Data curation (supporting); formal analysis (supporting); software (equal); validation (supporting); writing – review and editing (supporting). **Oliver Floerl:** Conceptualization (equal); funding acquisition (equal); writing – review and editing (supporting). **Andrew Jeffs:** Funding acquisition (supporting); supervision (supporting); writing – review and editing (supporting). **Anastasija Zaiko:** Conceptualization (equal); funding acquisition (equal); methodology (supporting); project administration (lead); supervision (lead); writing – review and editing (supporting).

## FUNDING INFORMATION

This work was supported by the New Zealand Ministry of Business, Innovation and Employment funding (CAWX1904—A Toolbox to Underpin and Enable Tomorrow's Marine Biosecurity System) and the Departmental Research Development Fund of the Institute of Marine Science, University of Auckland.

## CONFLICT OF INTEREST STATEMENT

The authors declare that they have no conflicts of interest.

### OPEN RESEARCH BADGES

This article has earned an Open Data badge for making publicly available the digitally‐shareable data necessary to reproduce the reported results. The data is available at https://doi.org/10.17608/k6.auckland.c.6242784.

## Data Availability

The environmental distances dataset used in this study (Tzeng, 2022c) is available as a University of Auckland Figshare collection (https://doi.org/10.17608/k6.auckland.c.5564757). The voyage‐related distances dataset is available as a University of Auckland Figshare collection (https://doi.org/10.17608/k6.auckland.c.6242784). Following best practices described by the Geoscience Papers of the Future initiative (Gil, David, et al., 2016), datasets are accompanied by ISO 19139 metadata (ISO, 2019), the R scripts are accompanied by OntoSoft metadata (Gil, Garijo, et al., 2016) and the computational workflows are described in ReadMe text files.
